# High Right Ventricular Afterload during Exercise in Patients with Pulmonary Arterial Hypertension

**DOI:** 10.3390/jcm10092024

**Published:** 2021-05-09

**Authors:** Mari Nishizaki, Aiko Ogawa, Hiromi Matsubara

**Affiliations:** 1 Department of Rehabilitation, National Hospital Organization Okayama Medical Center, Okayama 701-1192, Japan; 2 Department of Clinical Science, National Hospital Organization Okayama Medical Center, Okayama 701-1192, Japan; aikoao@gmail.com (A.O.); matsubara.hiromi@gmail.com (H.M.); 3 Department of Cardiology, National Hospital Organization Okayama Medical Center, Okayama 701-1192, Japan

**Keywords:** pulmonary arterial hypertension, pulmonary hemodynamic response to exercise, right ventricular afterload, exercise training

## Abstract

The right ventricle (RV) is more sensitive to an increase in afterload than the left ventricle (LV), and RV afterload during exercise increases more easily than LV afterload. Pulmonary arterial hypertension (PAH)-specific therapy has improved pulmonary hemodynamics at rest; however, the pulmonary hemodynamic response to exercise is still abnormal in most patients with PAH. In these patients, RV afterload during exercise could be higher, resulting in a greater increase in RV wall stress. Recently, an increasing number of studies have indicated the short-term efficacy of exercise training. However, considering the potential risk of promoting myocardial maladaptive remodeling, even low-intensity repetitive exercise training could lead to long-term clinical deterioration. Further studies investigating the long-term effects on the RV and pulmonary vasculature are warranted. Although the indications for exercise training for patients with PAH have been expanding, exercise training may be associated with various risks. Training programs along with risk stratification based on the pulmonary hemodynamic response to exercise may enhance the safety of patients with PAH.

## 1. Introduction

Pulmonary arterial hypertension (PAH), defined as resting mean pulmonary arterial pressure (mPAP) > 20 mmHg, pulmonary artery wedge pressure (PAWP) ≤ 15 mmHg, and pulmonary vascular resistance (PVR) ≥ 3 wood units in the absence of other causes of pre-capillary pulmonary hypertension (PH), is a cardiopulmonary disorder characterized by a progressive increase in PVR, resulting in right ventricular (RV) failure [1,2]. Although the pathological origin of the disease is in the pulmonary arterioles, secondary RV failure due to increased afterload is the main cause of exercise intolerance and a poor prognosis [1]. In patients with PAH, the RV adapts to the chronically increased afterload by increasing contractility and wall thickness at an early stage. Cardiac output (CO) may be limited during exercise (decreased contractile reserve), resulting in a decreased capacity for aerobic exercise. Consequently, RV dilatation and systolic dysfunction may occur, with the RV unable to adapt to a progressive increase in afterload. In the advanced stage of PAH, myocardial fibrosis and stiffening cause diastolic dysfunction, resulting in a reduced CO even at rest [3]. Inflammation of the RV may contribute to RV dysfunction and maladaptive remodeling [4]. PAH-specific therapy has improved the pulmonary hemodynamics at rest, exercise capacity, quality of life, and prognosis of patients in the last two decades [5,6,7]. The RV is more sensitive to an increase in afterload than the left ventricle (LV), and RV afterload during exercise increases more easily than LV afterload [8]. In the previous study, we found that exercise capacity was reduced and pulmonary hemodynamic response during incremental submaximal exercise testing was abnormal in most patients with PAH, even after hemodynamics at rest were improved with PAH-specific therapy [9]. In these patients, RV afterload during exercise could be higher, and exercise has the potential to promote myocardial remodeling. This review highlights the high residual RV afterload during exercise in patients with PAH whose hemodynamics at rest were significantly improved with PAH-specific therapy.

Exercise training is provided based on an appropriate exercise prescription, which is different from maximal exercise, and it is recognized as an adjunctive therapy for physically deconditioned patients with PAH recently. An increasing number of reports have indicated the efficacy of exercise training in the last decade [10,11,12]. Although adverse events could occur during training, only a few serious adverse events have been reported when low-intensity training was closely monitored at a specialized center [10]. However, the long-term effects of exercise training on the RV and the pulmonary vasculature are still unknown, and a training program has not been established for exercise training yet [10]. This review also focuses on training programs to enhance the safety of patients with PAH.

## 2. Acute and Chronic RV Responses to Exercise

At rest, the pulmonary circulation has a lower pressure and lower resistance system compared to the systemic circulation, due to the high compliance of the RV with its thin wall and reserve capacity for pulmonary circulation (dilatation and recruitment of pulmonary vasculature) [13]. As a result, the RV requires less energy than the LV to pump blood. The PVR decreases during exercise; however, this decrease is less than that of systemic vascular resistance because systemic circulation has a larger reserve capacity (vasodilation of the large vascular network for peripheral skeletal muscles) [8]. Therefore, the relative increase in RV afterload is greater than that in LV afterload. The stress on the RV wall is much higher than that on the LV wall, according to the Laplace formula, and the RV requires more energy than the LV to pump blood. The RV is more sensitive to an increase in afterload than the LV [14]. The RV demonstrated a high sensitivity to the change of afterload [15]. Even if RV injury is transient and repaired within a few days, when exercise is too hard and long, it may lead to RV remodeling and dysfunction in endurance athletes [16,17,18]. Exercise-induced acute RV dysfunction with no LV dysfunction was seen in endurance athletes [16]; RV inflammation without evidence of LV inflammation has been documented in rats with progressive PH [19].

## 3. Pathophysiology of Exercise Intolerance in Patients with PAH

**Figure 1:**
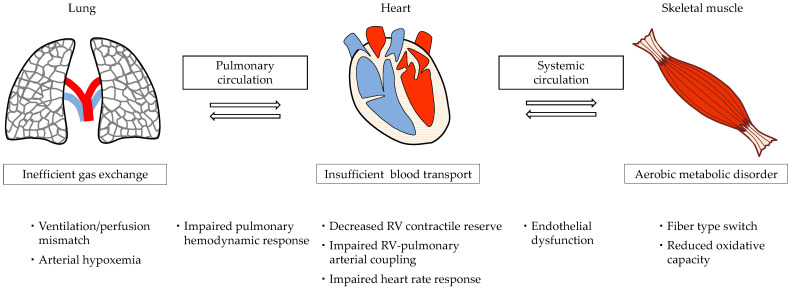
Schematic summary of the pathophysiology of exercise intolerance in patients with pulmonary arterial hypertension. Although pulmonary arterioles are the center of the pathological abnormality, an increase in RV afterload leads to secondary RV failure in patients with pulmonary arterial hypertension. Complicated central and peripheral dysfunctions relate to exercise intolerance. RV: right ventricular.

The energy for exercise is produced mainly by aerobic metabolism, and it relies on both central factors (lung, heart, and pulmonary circulation) and peripheral factors (skeletal muscle and systemic circulation) [20]. Exercise intolerance in patients with PAH is caused by the complicated mechanisms of central and peripheral dysfunctions (Figure 1). Abnormal proliferation of vascular cells in the small pulmonary arterioles along with imbalance of pulmonary vasodilators and vasoconstrictors results in a reduction in the reserve capacity of pulmonary circulation [21], which could cause an abnormal hemodynamic response to exercise. Abnormal pulmonary hemodynamic responses can result in inefficient gas exchange. Hypoperfusion of ventilated alveoli increases the ventilation/perfusion mismatch, resulting in exertional dyspnea [22]. A reduced pulmonary capillary bed can cause arterial hypoxemia, leading to exertional dyspnea [22]. RV contractility increases at rest, whereas RV contractile reserve decreases [23]. The RV-pulmonary arterial (PA) coupling indicates the efficiency of energy transfer from the RV to the PA based on the notion that the right heart and the pulmonary vasculature are not separate systems, but continuous cardiovascular units [24]; this efficiency is calculated as the ratio between RV end-systolic elastance (RV contractility) and PA elastance (RV afterload) [25]. RV–PA coupling was impaired during exercise in patients with PAH because of an increased RV afterload without a sufficient increase in RV contractility to compensate for it [23]. LV diastolic dysfunction due to ventricular septal displacement also occurs due to the increased RV afterload [3]. Insufficient blood transport to cater to the oxygen demand leads to exertional fatigue and dyspnea. The baseline stroke volume and heart rate response were independent determinants of exercise capacity in patients with PAH [26,27]. Patients display a low proportion of type I muscle fibers and reduced muscle oxidative capacity [28], resulting in aerobic metabolic disorders. The aerobic capacity of the skeletal muscle is reduced, and the early occurrence of anaerobic metabolism leads to exertional fatigue. In addition, early lactic acidosis stimulates breathing, resulting in dyspnea [22]. The maximum oxygen uptake was correlated with the strength of the quadriceps, and oxygen uptake at the anaerobic threshold was correlated with muscle oxidative capacity [28]. Peripheral endothelial dysfunction may result in impairment of systemic circulation [29], leading to exertional fatigue.

## 4. Pulmonary Hemodynamic Response to Exercise in Normal Subjects

In a systematic review of 1187 healthy patients, a modest decrease in PVR was observed with an increase in CO during exercise, which led to a modest increase in mPAP [30,31,32]. The mPAP during exercise did not exceed 30 mmHg at CO < 10 L·min^−1^ [33]. An mPAP/CO slope or an mPAP/workload slope may be more suitable to evaluate the pulmonary hemodynamic response to exercise than the mPAP alone [34]. Lewis et al. showed that mPAP/CO in healthy participants ranged from 0.8 to 2.5 mmHg·min·L^−1^ in their review [31], and Naeije et al. indicated that mPAP/CO ≈ 1 mmHg·min·L^−1^ in young adults and mPAP/CO ≈ 2.5 mmHg·min·L^−1^ in old adults [35].

## 5. Pulmonary Hemodynamic Response to Exercise in Patients with PAH

Pulmonary hemodynamic responses are impaired in patients with PAH. Although PH was defined as resting mPAP ≥ 25 mmHg or mPAP during exercise ≥ 30 mmHg, the exercise criterion was eliminated because of the lack of reliable data [36]. However, exercise could be useful in assessing the reserve capacity of pulmonary circulation and RV contractility. While a rise in resting mPAP is a late event in the course of PAH, exercise-induced PAH could be an indicator of the early and mild phases of PAH [30]. An increase in RV contractility could not sufficiently compensate for an increased RV afterload, resulting in reduced RV-PA coupling during maximum exercise in patients with exercise-induced PH [37]. There are three different definitions of exercise-induced PH. First, the combined parameters of mPAP at peak exercise > 30 mmHg and TPR at peak exercise as a single point of mPAP-CO ratio > 3 mmHg·min·L^−1^ are used to define exercise-induced PH [38], which reduced false positives in healthy subjects compared to the previous criterion defined as only mPAP during exercise > 30 mmHg [39]. Second, the multipoint mPAP/CO slope is used. The slope utilizes multiple mPAP-CO ratios during exercise (4–5 points), and a slope > 3 mmHg·min·L^−1^ is defined as exercise-induced PH [31]. Third, the two-point mPAP/CO slope is used: this slope is calculated as the change in mPAP from rest to peak exercise divided by the change in CO from rest to peak exercise, and a slope > 3 mmHg·min·L^−1^ is considered abnormal [40].

Pulmonary hemodynamic responses to exercises in patients with PAH are shown in Table 1 [9,30,31,41,42,43,44,45,46,47,48]. At present, drugs targeting the prostacyclin, endothelin, and nitric oxide pathways are available, and upfront combination therapy is recommended in patients with PAH [1]. Randomized clinical trials have shown the efficacy of this therapy in exercise capacity and time to clinical worsening [49]. However, the pulmonary hemodynamic response would remain abnormal even after pulmonary hemodynamics at rest could be improved with PAH-specific therapy (Table 1 [9,47,48]).

In a study using monocrotaline-induced rats with PH, exercise capacity was improved with moderate-intensity exercise when CO was preserved; however, exercise induced RV inflammation and pulmonary vascular remodeling, leading to a reduction in survival rate in progressive PH rats on a higher dose of monocrotaline [19]. While the relationship between chronic RV wall stress and RV inflammation has not yet been clarified, RV maladaptation to an increased afterload could lead to poor outcomes. In addition, this study suggests that the effect of exercise could differ depending on disease severity [19]. Exercise may promote disease progression, at least in severely ill patients.

**Figure 2:**
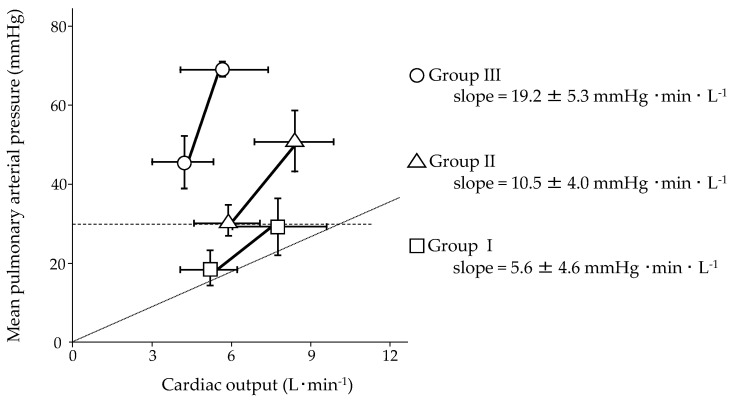
Mean pulmonary arterial pressure (mPAP)/cardiac output (CO) slope during submaximal exercise testing in 32 patients with pulmonary arterial hypertension (PAH) stratified into three groups based on mPAP at rest. All the patients were clinically stable with PAH-specific combination therapy. The figure was generated from our original data recently published [9]. Group I (square) indicates patients with resting mPAP < 25 mmHg, Group II (triangle) indicates patients with 25 mmHg ≤ resting mPAP < 40 mmHg, and Group III (circle) indicates patients with resting mPAP ≥ 40 mmHg. Straight line indicates mPAP/CO slope of each group during exercise testing. The broken line represents resting mPAP of 30 mmHg, and the dotted line represents the mPAP/CO slope of 3 mmHg·min·L^−1^.

**Figure 3:**
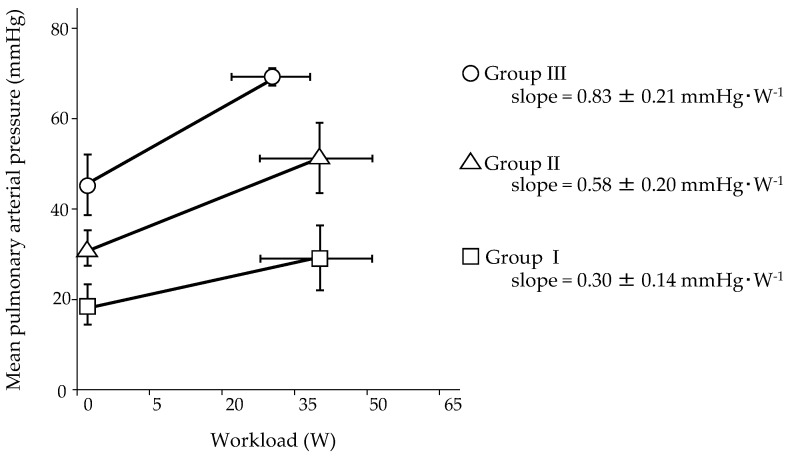
Mean pulmonary arterial pressure (mPAP)/workload slope during submaximal exercise testing in 32 patients with pulmonary arterial hypertension stratified into three groups based on mPAP at rest. All the patients were clinically stable with PAH-specific combination therapy. The figure was generated from our original data recently published [9]. Group I (square) indicates patients with resting mPAP < 25 mmHg, Group II (triangle) indicates patients with 25 mmHg ≤ resting mPAP < 40 mmHg, and Group III (circle) indicates patients with resting mPAP ≥ 40 mmHg. Straight line indicates mPAP/workload slope of each group during exercise testing.

An impaired pulmonary hemodynamic response to exercise could be one of the keys to clinical deterioration. We clarified the relationship between baseline hemodynamics and the mPAP/CO slope during exercise in patients with PAH by reassessing our original and recently published data on 32 patients, whose hemodynamics at rest were moderately to severely impaired before treatment [9]. After PAH-specific combination therapy, patients were clinically stable and were classified a functional class I or II according to the World Health Organization (WHO). Incremental submaximal exercise testing with right heart catheterization was performed using a cycle ergometer. The patients were classified based on resting mPAP (Group I: resting mPAP < 25 mmHg, Group II: 25 mmHg ≤ resting mPAP < 40 mmHg, and Group III: resting mPAP ≥ 40 mmHg), and their two-point mPAP/CO slopes were assessed (Figure 2). During the exercise test of the three groups, it was found that the mPAP increased modestly with an increase in CO, which was reduced compared to healthy subjects (low pressure, low flow) in Group I [30,31]. The mPAP increased significantly in Group II; however, the increase in CO was reduced (high pressure, low flow) in this group. In Group III, the mPAP increased significantly, and CO increased slightly (very high pressure, very low flow). The mPAP/CO slope of Group I was nearly parallel, with a slope of 3 mmHg·min·L^−1^. The slope of Group II was steeper than that of Group I, and the slope of Group III was steeper than that of Group II. Thus, the pulmonary hemodynamic response in Group I was near normal, and the impairment of the pulmonary hemodynamic response in Group III was more severe than that in Group II. This result suggests that the RV afterload was much higher in Group III, resulting in a greater increase in RV wall stress than in the other two groups. The mPAP/workload slopes for the three groups are shown in Figure 3. Hasler et al. investigated the pulmonary hemodynamic response in patients with PAH (n = 54) or distal chronic thromboembolic PH (n = 16), 35% of whom were undergoing PAH-specific combination therapy [47]. Although resting mPAP was 34 mmHg, mPAP/CO slopes were steep (multipoint mPAP/CO slope was 14.2 mmHg·min·L^−1^ and two-point mPAP/CO slope was 17.0 mmHg·min·L^−1^) (Table 1), and the mPAP/CO slopes were predictive of 3-year transplant-free survival. This result might substantiate the aforementioned detrimental effect of exercise in progressive PH rats with RV maladaptation to increased afterload [19].

## 6. The Efficacy and Safety of Exercise Training in Patients with PAH

Recently, an increasing number of studies have shown the efficacy of exercise training on molecular, functional, physiological, and psychological factors [10,11,12]. The first randomized clinical trial for rehabilitation in patients with PAH was reported by Mereles et al. in 2006 [50]. Patients with PAH (n = 24) and inoperable chronic thromboembolic PH (n = 6) were randomized to a sedentary control group or to a 3-week in-hospital and 12-week home-based training group. The program consisted of aerobic exercise training, muscle training, and respiratory training. After 15 weeks, peak oxygen uptake significantly increased (from 13.2 to 15.4 mL·min^−1^·kg^−1^) in the training group; however, the sedentary control group showed no significant changes. The 6-min walk distance also significantly increased (96 m) in the training group. In addition, five of the eight subscale scores by the 36-Item Short Form Health Survey significantly increased. Similar randomized clinical trials have been conducted thereafter. Aerobic exercise training for 3–15 weeks significantly improved exercise capacity, physical activity, fatigue severity, and quality-of-life scores [48,51,52,53,54]. In the 2015 ESC/ERS Guidelines for the Diagnosis and Treatment of Pulmonary Hypertension, supervised exercise training is considered for stable patients with PAH receiving medical therapy (class of recommendation IIa, level of evidence B) [1].

In a large prospective cohort study, adverse events, such as syncope, pre-syncope, supraventricular tachycardia, and respiratory infection, occurred in 13.6% of 183 patients, although most of them were not directly caused by exercise training, and clinical deterioration was not detected during hospitalization [55]. However, there remains a lack of evidence indicating the long-term safety of exercise training. Randomized clinical trials investigating disease progression and survival in patients with PAH are necessary.

The mechanisms underlying the improvement of exercise capacity with exercise training could be multifactorial and have not yet been clarified. De Man et al. indicated that exercise training increased capillarization and oxidative enzyme activity, which are related to the improvement of quadriceps endurance [56]. While the main mechanism in the improvement of exercise capacity is supposed to be the improvement of peripheral factors, improvement of central factors might also be involved. In a study using monocrotaline-induced PH rats with preserved CO, moderate-intensity exercise did not cause disease progression, and exercise capacity was improved with enhanced RV capillarization [19]. However, there is little clinical evidence indicating the favorable effects of exercise training on the central factors in human studies. A single study demonstrated improvement in pulmonary hemodynamics by exercise training for 15 weeks [48]. Long-term observation of the effects of exercise training on the pulmonary vasculature and RV remodeling is necessary.

## 7. Exercise Training Program Based on Risk Stratification to Enhance Safety and Efficacy

The optimal time to start, the appropriate style, frequency, intensity, and duration of exercise training have not yet been established [10]. Exercise training could not be safe for patients with severe PAH when it is too strong or long; on the other hand, its efficacy could not be sufficient for patients with mild PAH when it is too weak or short. A training program prescribed with minimal risks and maximal benefits for each patient is ideal. The indications for exercise training in patients with PAH are expanding. Since the various risks of exercise training in patients with PAH cannot be ignored, risk management of exercise training is mandatory. In a statement by the American Heart Association, age, presence of heart disease, clinical characteristics (New York Heart Association functional class, exercise test results, previous episode of primary cardiac arrest), and intensity of exercise are major factors that affect the risk of exercise [57]. Based on these factors, subjects were stratified into four classes: Class A: apparently healthy individuals; Class B: patients with stable cardiovascular diseases with a low risk for complications with vigorous exercise, with the risk being slightly greater than that for apparently healthy individuals; Class C: those at moderate-to-high risk for cardiac complications during exercise and/or unable to self-regulate activity or to understand recommended activity level, and Class D: those with an unstable disease with activity restriction. Activity guidelines, the need for supervision, and the level of monitoring required during exercise were provided for each class.

It is clear that aerobic exercise training and muscle training are beneficial with little risk to patients with PAH whose hemodynamic response to exercise is normalized or mildly impaired (Group I in Figure 2 and Figure 3). Exercise training requires close monitoring at a center well experienced in both treatment and rehabilitation of PAH in the early phase; however, it could be transferred to home-based training with regular evaluation at the center. Patients may be more comfortable with home-based training, which may also contribute to medical cost reduction. In patients with a severely impaired hemodynamic response (Group III in Figure 2 and Figure 3), interval low-intensity training with close monitoring at a specialized center could be safe in the short term; however, repetitive exercise training, even if it is low-intensity, can potentially cause myocardial remodeling, leading to long-term clinical deterioration. The overall treatment goal in patients with PAH is the acquisition of a low-risk status, which is associated with exercise capacity, quality of life, RV function, and mortality risk [1]. It was shifted from short-term functional change to improvement of long-term outcomes at the 5th World Symposium on PH in 2013 [58]; exercise training must be performed with this treatment goal. We should not recommend exercise training to patients who could deteriorate in the long term. However, patient-based efficacy cannot be neglected. The value of exercise training can differ between physicians and patients. For patients, temporary improvement of symptoms, exercise capacity, or quality of life might count. Exercise training in patients with moderately impaired pulmonary hemodynamic response to exercise (Group II in Figure 2 and Figure 3) should be performed cautiously. It is worth noting that all patients in Figure 2 and Figure 3 were classified as WHO functional classes I or II. Individual training programs should be prescribed with appropriate intensity based on subjective symptoms and objective evaluation by cardiopulmonary exercise testing before rehabilitation starts. However, this is difficult, especially when hemodynamic impairment is severe. Resting mPAP may be a predictor of abnormal pulmonary hemodynamic responses to exercise. We hope that a training program that contributes to the best outcome will be established in the near future.

## 8. Conclusions

Even after hemodynamics at rest are improved with PAH-specific therapy, RV afterload during exercise could be high due to residual impairment of the pulmonary hemodynamic response in patients with PAH. The short-term efficacy of exercise training was established; however, the RV is sensitive to increased afterload, and even low-intensity repetitive exercise training with a high RV afterload could carry a risk of promoting myocardial maladaptive remodeling in the long term. Training programs along with risk stratification may enhance the safety of exercise in patients with PAH.

## Figures and Tables

**Table 1:** Pulmonary hemodynamic response to exercise.

First Author, Year [Ref.]	n	PAH, %	Age, y	WHO fc, n I/II/III/IV	Exercise Protocol	Workload, W	mPAP, mmHg		CO, L·min^−1^ (CI, L·min^−1^·m^−2^)		mPAP/CO Slope, mmHg·min·L^−1^ (mPAP/CI Slope, mmHg·min·L^−1^·m^−2^)	PAH-Specific Therapy
							Rest	Ex	Rest	Ex		
Tolle, 2008 [30] Lewis, 2013 [31]	16	0	46 ± 15	—	Cycle	156 ± 43	14 ± 3	27 ± 4	5.8 ± 1.0	15.5 ± 3.2	NA	—
Janicki, 1985 [41]	9	56	NA	NA	Treadmill	N/A	43 ± 16	81 ± 16	(1.9 ± 0.3)	(5.0 ± 1.5)	13.4 ± 9.5 ^#^	none
Blumberg, 2002 [42]	16	63	NA	0/5/11/0	45°-Cycle	25 or 50	45 ± 8	70 ± 13	3.7 ± 1.0	5.8 ± 2.4	NA	none or aerosolized iloprost
Castelain, 2002 [43]	7	100	46 ± 14	0/0/7/0	Supine-Cycle	0–60	52 ± 8	NA	(2.6 ± 0.6)	NA	(13.1) ^‡^	intravenous prostacyclin
Provencher, 2008 [44]	42	100	48 ± 13	0/22/20/0	Supine-Cycle	0–40	52 ± 14	76 ± 17	(2.9 ± 0.7)	(4.3 ± 1.3)	(21.5 ± 15.2) ^‡^	ERA and/or intravenous prostacyclin
Chemla, 2013 [45]	12	58	45 ± 14	NA	Supine-Cycle	0–60	57 ± 9	75 ± 10	4.4 ± 1.4	6.1 ± 2.1	11.7 ± 5.7 ^#^	NA
Chaout, 2014 [46]	55	100	54 ± 16	8(I/II)/32/15	Supine-Cycle	20 *	52 ± 13	70 ± 17	(2.0 ± 0.6)	(2.8 ± 1.1)	NA	NA
Hasler, 2016 [47]	70	77	65 *	20(I/II)/35/15	Supine-Cycle	30 *	34 *	55 *	5.2 * (2.8 *)	6.4 * (3.5 *)	14.2 *^,#^ 17.0 *^,##^	dual: 31%, triple: 4%
Ehlken, 2016 [48]	41	63	57 ± 15	1/6/30/4	Supine-Cycle	72 ± 23	38 ± 12	58 ± 18	5.1 ± 1.8 (2.7 ± 0.9)	9.1 ± 2.3 (4.7 ± 1.1)	NA	mono: 35%, dual: 55%, triple: 10%
Nishizaki, 2020 [9]	32	100	33 ± 10	7/25/0/0	40°-Cycle	38 ± 11	28 ± 11	46 ± 17	(3.7 ± 0.9)	(5.4 ± 1.2)	10.0 ± 6.7 ^##^	dual: 41%, triple: 59%

Data are presented as mean ± standard deviation, range, median *, number, or %. PAH: pulmonary arterial hypertension; WHO fc: World Health Organization functional class; mPAP: mean pulmonary arterial pressure; CO: cardiac output; CI: cardiac index; Ex: exercise; ERA: endothelin receptor antagonists; #: multipoint mPAP/CO slope; ##: two-point mPAP/CO slope; ‡: multipoint mPAP/CI slope.

## Data Availability

The datasets of this study are available on reasonable request to the corresponding author.
